# Implications of Nutrient Fate and Transport Following Nanopesticide Applications in Agricultural Field Plots in Central Kentucky

**DOI:** 10.3390/toxics13090758

**Published:** 2025-09-06

**Authors:** William Rud, Manuel D. Montaño, Daniel N. Miller, Wayne Sanderson, Carmen Agouridis, Brianna F. Benner, Tiffany L. Messer

**Affiliations:** 1 Biosystems and Agricultural Engineering Department, University of Kentucky, 128 Barnhart, Lexington, KY 40506, USA; wayne.sanderson@uky.edu (W.S.); carmen.agouridis@uky.edu (C.A.); tiffany.messer@uky.edu (T.L.M.); 2 Department of Environmental Sciences, Western Washington University, Bellingham, WA 98225, USA; montanm2@wwu.edu (M.D.M.); bennerb3@wwu.edu (B.F.B.); 3 United States Department of Agriculture, Agricultural Research Service, Lincoln, NE 68583, USA; dan.miller@usda.gov

**Keywords:** nanopesticides, emerging contaminants, copper hydroxide, agroecosystems, nutrients, runoff, agriculture

## Abstract

The potential benefits of nanopesticide use over standard pesticides include more precise application at reduced application rates, lower premature degradation, and decreased direct impacts to target organisms. However, field scale investigations of the fate and transport of common nanopesticides such as copper (II) hydroxide and imidacloprid combinations, remain limited. A field study evaluating nano-scale copper (II) hydroxide (Cu), standard imidacloprid (I), nanoimidicloprid (NI), and nano-scale copper (II) hydroxide and imidacloprid (CuNI) compared to control (C) plots was conducted using thirty 14.6 m^2^ field plots to determine the impacts of nanopesticide applications on nutrient cycling and quantify the persistence of copper (II) hydroxide in soil and surface runoff during the growing season. Soil samples were taken at the beginning and end of the growing season, while water quality runoff samples were collected following eleven rainfall events. Ammonium concentrations in runoff decreased in CuNI plots by 1.74 mg N/L, while total nitrogen concentrations in runoff increased by 1.29 mg N/L in Cu plots compared to CuNI plots. Runoff orthophosphate concentrations decreased in CuNI treatments compared to all other pesticide treatments by 1.37, 1.32, and 1.30 mg P/L in Cu, I, and NI plots, respectively. Significant increases in soil copper concentrations were also observed in all plots receiving Cu. These findings emphasize the potential biogeochemical implications of using these nanopesticides on nutrient cycling in agroecosystems.

## 1. Introduction

With a growing need for more efficient crop production, practical pesticide use is essential to prevent damage from insects, bacteria, fungus, and other plants [1]. Pesticide use increased 11% per year from the 1950s until the early 2000s worldwide marking a higher need for agricultural products [2], but has since plateaued at about 2.7 billion kg per year as of 2020 [3]. Pesticides have previously been administered using bulk application methods, which has resulted in some cases in negative environmental implications and human health concerns [4,5,6]. Pesticides have a long history of being transported in runoff as non-point source pollutants entering surface waters, often resulting in negative impacts to non-target species [4,7]. To minimize these implications, the development of engineered nanoparticles (ENP) has been explored as a potential solution to improve crop production while reducing the amounts of active pesticide ingredients applied [8].

There are three criteria required for a product to be classified as a nanopesticide: the particle is under 100 nm in size, the nano prefix is attached to the product, and the product is novel because of the ingredients size [9,10]. These pesticides have been marketed with new properties anticipated to provide higher efficacy at lower application rates due to their slower release rates, reduced toxicity, and unique particle behavior [11,12]. The unique properties of nanosized materials have been theorized to impact nutrient runoff rates by altering nutrient cycles, including nitrogen (N), phosphorus (P), and carbon (C), through physical and biogeochemical processes [13,14]. Nanoparticles also have lower active ingredient amounts, which provide the potential to limit non-point source pollution of these pesticides impacting waterways [12,13,14].

Copper (II) hydroxide utilizes the antimicrobial properties of dissolved copper to reduce development of fungi on agricultural crops [15,16]. Bulk copper pesticides (non-nano formulations) have been shown to impact soil microbial health after application, specifically affecting P cycling [17], N cycling [16], and bioavailability of dissolved organic carbon (DOC) [15]. Cu(OH)_2_ and CuO have been found to impact soil microbial health after application [16]. For instance, Cu(OH)_2_ resulted in reduced bacterial and fungal communities following application [17]. Similarly, CuO has been observed to acutely decrease soil denitrification, increase N_2_O production, decrease electron transport system activity, and alter the denitrifying functional genes [16]. These describe all negative changes to the necessary functions of the soil microflora, which will potentially lead to increased nutrient release from soils.

In contrast, imidacloprid, a neonicotinoid insecticide, is regularly applied via seed treatment, foliar, soil application, or stem application for control of biting and sucking insects by impacting central and peripheral nervous systems of insects due to its systemic properties [18,19]. Imidacloprid has been shown to undergo breakdown processes in plants, vertebrates, invertebrates, soil, and water by several physicochemical and metabolic processes; however, degradants of imidacloprid are primarily formed via photolysis in soil and surface runoff [3]. Currently, there remains an absence of explorations of interactions between imidacloprid and Cu(OH)_2_. However, Zhang et al. (2019) observed reduction in bioavailable thiacloprid and adaptations in the microbial community composition following application of both Cu(OH)_2_ and thiacloprid [20].

Nano-formulations of copper (II) hydroxide have been developed, but to date there have been limited studies on fate, transport, and biogeochemical implications of copper (II) hydroxide nanopesticides in agricultural field applications and in combination with agricultural relevant insecticides. A recent review on nano-enabled pesticides emphasized the need for field testing of nanopesticides to assess implications on fate and transport of the pesticides and nutrients under relevant field and rapidly changing climate conditions and potentially associated application risks [12].

Therefore, the goals of this study were to assess implications of the nano-formulation of copper (II) hydroxide in combination with potential co-applied insecticides on biogeochemical cycling in a field-scale agroecosystem and to determine nutrient transport via runoff to downstream ecosystems during the growing season as detailed in Rud [21]. The specific aims of this study were: 1. Determine the impact of nanopesticides formulations of copper (II) hydroxide and imidacloprid (an agricultural relevant insecticide) on nutrient cycling in a field-scale agricultural application site. 2. Quantify the persistence of copper (II) hydroxide in soil and surface runoff throughout the growing season.

## 2. Materials and Methods

### 2.1. Site Characteristics

**Figure 1:**
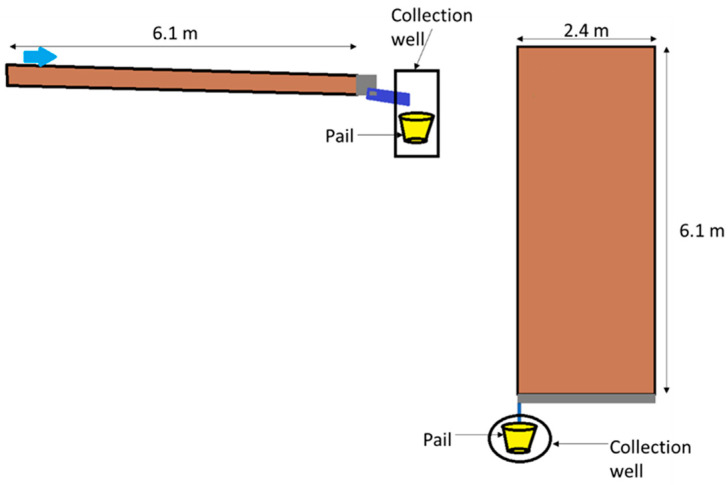
Diagram of plot construction.

Agricultural field plots were located on North Farm, which is owned by the University of Kentucky in Lexington, Kentucky, USA (38.12144° N, 84.48714° W), and evaluated during the growing season of 2022. The soil was classified as Maury Silty Loam, additional information on the initial soil conditions is in Supplemental Information Table S1 [22]. Plots construction is detailed in Edwards et al. [23] and shown in Figure 1. In summary, the field site comprised 30 individual 2.4 m by 6.1 m plots with a 3% slope. The plots were contained using impermeable steel borders driven 10 cm into the soil with 10 cm remaining above the soil surface along three edges to direct water flow to the down gradient point with the final edge comprising a stainless gutter and PVC pipe to direct runoff into a 14 L polyethylene pail for sample collection. Collection pails were covered to prevent dilution from rainwater and degradation from sunlight.

A weather station was installed at the research site and comprised a HOBO RG2 Data Logging Rain Gauge (Onset, Bourne, MA, USA) and HOBO Pendant MX Temperature/Light Data Logger (Onset, Bourne, MA, USA). The sensors recorded temperature and light at the field plots in 15 min intervals, along with rainfall amount and intensity throughout the study. In the event of equipment malfunction, data were collected from the National Weather Service Lexington Blue Grass Airport (38.03° N, 84.61° W) weather station [24] approximately 20 km from the field site. Additional details of materials and methods used for this study can be found in Rud [21].

### 2.2. Field Experiments

To prepare the soil for planting and remove fescue plots were rototilled multiple times throughout the spring of 2022 prior to planting and pesticide applications. The selected crop for the plots was organic Jack Be Little ornamental pumpkins (Cucurbita pepo; Urban Farmer, Westfield, IN, USA) and was selected for its use for all applied pesticides and growing season period. Prior to planting, soil was tilled into two columns with five rows of mounds, and three seeds were added to each mound [25]. Planting was completed on 19 July 2022.

**Figure 2:**
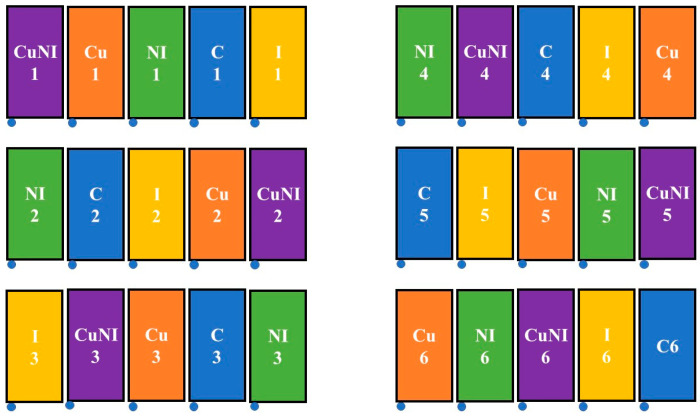
Field plot treatment design. Treatments included: C—control (blue), Cu—copper nanopesticide (orange), CuNI—copper nanopesticide and nano-imidacloprid (purple), NI—nano-imidacloprid (green), and I—imidacloprid (yellow).

Five pesticide treatments were assessed at the field plots in replicates of six. The treatments were as follows: control with no pesticide application (C), nano-imidacloprid (NI), copper nanopesticide (Cu), standard imidacloprid (I), and copper nanopesticide with nano-imidacloprid (CuNI). Treatments were blocked as shown in Figure 2. Commercial conventional formulation (Tree & Shrub BioAdvanced, Cary, NC, USA) and nano-imidacloprid (company non-disclosure agreement) applications were applied on 14 July 2022, as a pre-planting soil application using guidelines provided by the manufacturers. The copper (II) hydroxide nanopesticide (Kocide 3000, Certis, Columbia, MD, USA) was applied as a foliar application to emerging leaves on 2 August 2022. All applications followed application guidelines on pesticide containers and were performed using 3.7 L. hand pump sprayers with an application height of 10 cm. Imidacloprid was applied at a mixing rate of 170 mL of product per plot diluted into 1.9 L of tap water, while nano-imidacloprid was added at a mixing rate of 3.1 mL product per plot diluted into 1.9 L of tap water. The Cu application was applied at 2.08 g of product per plot diluted into 1.9 L of tap water. The mass of applied pesticides and nutrient residuals per plot are shown in Table 1.

### 2.3. Sample Collection

Runoff samples were collected within 12 h following each rainfall event that produced rainfall in half of the rainfall collection pails at the bottom of the plots during the study period, which spanned 9 July 2022 to 31 August 2022. Samples were collected by treatment to avoid cross contamination. During collection, runoff collection pails were removed from the wells at the bottom of each field plot, mixed, and the volume was recorded. Samples were collected for organic pesticides using 20 mL glass vials, metals using 60 mL amber polypropylene bottles, and nutrients (including N, P, and C species) using a 250 mL polypropylene bottle. Sample containers were placed into coolers with icepacks to reduce microbial activity and sunlight exposure during transport back to the *meso*Lab (University of Kentucky, Lexington, KY, USA) for processing and analysis/shipping. The nutrient samples were filtered through a 0.7 µm glass fiber filter with exception to total Kjeldahl nitrogen (TKN)-N, and sulfuric acid was added to lower the pH to <2. An YSI EXO multiparameter sonde (Yellow Springs, OH, USA) was also used at the field sites to record runoff water temperature (°C), specific conductivity (µS/cm), and pH.

### 2.4. Nutrient Analysis

Samples were taken from the 250 mL polypropylene bottle and divided for respective sample preservation prior to assessments. All water N, P, and C species assessments were performed in the *meso*Lab using either a Seal Analytical AQ400 Discrete Analyzer (Mequon, WI, USA) or Shimadzu TOC-VCPN Total Organic Carbon Analyzer (Kyoto, Japan) with the following EPA methods: nitrate-N (NO_3_-N; EPA-126-C Rev. 2), orthophosphate-P (PO_4_-P; EPA-145-C Rev 1), ammonium-N (NH_4_-N; EPA-129-C Rev. 3), total Kjeldahl nitrogen-N (TKN; EPA-111-C Rev. 1A), and DOC (EPA Method 415.1).

### 2.5. Soils Analysis

Soil samples were collected pre-planting and post-harvest to assess potential changes in a suite of soil nutrients over the growing season. Sample containers were filled by taking cores at 15 to 20 cm depths from 10 locations in each plot. Cores were composited and transported to the University of Kentucky Regulatory Services Soil Testing Lab (Lexington, KY, USA). A routine soil test was performed for P, potassium (K), calcium (Ca), magnesium (Mg), zinc (Zn), pH, and cation exchange capacity (CEC) based on Soil Analysis Handbook of Reference Methods. Copper (Cu), aluminum (Al), and iron (Fe) concentrations were analyzed using the Mehlich III method from Soil Analysis Handbook of Reference Methods [26]. Total nitrogen (TN) was based on dried soil combustion in a LECO combustion instrument for %N produced, while organic matter was analyzed based on dried soil combustion in a LECO combustion instrument for %C produced based on Soil Analysis Handbook of Reference Methods [26].

### 2.6. Metals Analysis

All copper (II) hydroxide sample assessments were performed at Western Washington University (WWU) at the Institute of Environmental Toxicology and Chemistry. Dissolved trace metals were analyzed according to EPA Method 200.8. Briefly, samples were filtered using a 20 mL plastic syringe through a 0.45 µm nylon syringe filter and then diluted 1:10 in 2% nitric acid (Fisher, trace metal grade). A suite of external calibration standards was prepared using a mixed metal standard (ICP-MS-200.9-CAL-1, Accustandard, New Haven, CT, USA) to produce a range of concentrations from 1 ng/L to 1 mg/L. An internal standard was prepared (ICP-MS Internal STD Mix, Accustandard) at a concentration of 10 µg/L (Li-6, Sc, Y, In, Ho, Tb, Bi). Samples were then analyzed using an Agilent 7900 ICP-MS (Agilent Technologies, Santa Clara, CA, USA) operating in no-gas and in helium (He) gas collision mode. Typical operating parameters are listed in Supplementary Materials Table S2.

### 2.7. Statistical Analyses

Statistical analyses were completed for all water quality analytes concentrations based on treatments, blocking, and events using a one-way ANOVA with a statistical significance of α = 0.05. Normality and homogeneity of variance tests were conducted prior to the ANOVA and were viewed acceptable for field study. The natural log of the concentrations was used for mixed ANOVA/Tukey’s honest significant differences (HSD) for treatments. Additionally, the difference in soil physicochemical properties between the start of the growing season and the end were evaluated using One-way ANOVA/Tukey’s honest significant difference (HSD) by treatment. Physicochemical properties were not transformed for reporting means. Statistical analyses were conducted in SAS Studio 3.82 (SAS Institute Inc., Cary, NC, USA).

## 3. Results and Discussion

### 3.1. Rainfall Events

In total, 11 rainfall events were sampled with all events analyzed for N, P, and C species and events 3, 5, 6, 7, and 8 analyzed for total Cu in runoff samples (Table 2). In summary, the largest intensity events were events 4 and 7 with an average rainfall intensity of 57 mm hr^−1^. Event 5 was recorded as the largest event in depth with 32.6 mm of rainfall. Previous research has stated that higher intensity rainfall is related to higher loads of N and P losses with vegetative cover impacting runoff [27]. The largest span between rainfall events was between events 10 and 11 at nineteen days. These event dry-wet cycles have been shown to increase inorganic soil concentrations through mineralization and respiration when other microbial growth conditions are met [28,29].

### 3.2. Nitrogen Transport

NO_3_-N, NH_4_-N, TKN, and TN concentrations exported from each plot by event were evaluated with no significant differences across the treatment groups observed for NO_3_-N or TKN (Table 3). However, significant differences were observed in NH_4_-N concentrations, where less NH_4_-N was released in the CuNI treatments (*p* = 0.0458) compared to the C treatments resulting in a 1.74 ± 1.22 mg N/L decrease between treatments (Table 3). While increased amounts of N were applied on the I, NI, and CuNI plots (Table 1), loss of nitrogen in the NH_4_-N form does not align with potentail breakdown products of these pesticides, which would breakdown primiarily into NO_3_^−^ [30]. This presents a potential synergistic effect of the pesticides to NH_4_-N leaving the plots [31]; however, microbial data is needed to validate this hypothesis. Additionally, significant differences were observed in TN concentrations between plots, where the Cu plots had significantly more TN released compared to CuNI (*p* = 0.0285) resulting in 1.29 ± 1.08 mg N/L difference in water concentrations. Additional graphs of treatment and event with treatment box plots can be found in Supplementary Materials Figures S1 and S2, which exhibit normalized average concentrations leaving the plots across the entire season.

**Figure 3:**
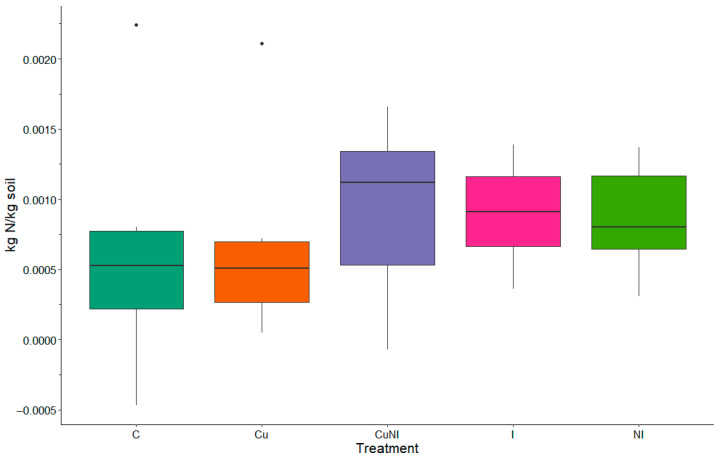
Difference in soil TN (kg N/kg soil) between pre-applications at the beginning of the growing season and end of the growing season by treatment (n = 5).

In the previous study completed by Williams and Edwards [32], fertilizer applications were applied, and the soil was not tilled prior to planting at the plots used in our study. This resulted in overall lower N species concentrations in this experiment. For example, average NO_3_-N concentrations leaving the plots by event for all treatments and events was 0.013 kg/ha, which was lower than previously reported from the same plots used as controls during an experiment in 2017, where 0.07 kg/ha was reported [32]. Similarly, 0.01 NH_4_-N kg/ha was observed in this study in contrast to the previous study using these plots, which observed 0.09 kg/ha [32]. Once again, TKN concentrations exported from the plots were lower at 0.013 kg/ha in comparison to 0.24 kg/ha previously observed [32]. Average TN exported from the plots was 0.029 kg/ha in this study compared to 0.33 kg/ha in the previous study [32]. This difference in N species export was likely due to N fertilizer not being applied to the plots based on pre-planting soil assessments at the beginning of the growing season (Figure 3). N observations in this study were instead more like Lentz and Westermann [31], which similar to our study, only used a rototiller to disturb the soil prior to planting crops at the initiation of the study.

### 3.3. Impacts to Soil Nitrogen Concentrations

No significant differences were observed between the beginning and end of the growing season for TN in soil between treatments (Figure 3). The overall average TN had a 0.08 ± 0.06% positive difference between the beginning and end of the growing season; meaning there was less TN in the soil following the growing season similar to observations by Davis et al. [33]. In a previous study completed by Davis et al. [33], fertilizer applications at unique rates including 0, 60, and 120 kg N ha^−1^ yr^−1^ were applied to various plots across Illinois, Kentucky, Nebraska, New Jersey, and Virginia to assess impacts on soil C and N concentrations with no impacts found across all fertilizer rates. Further, our observations were similar to observations of Das et al. [34], which did not observe significant impacts on gross nitrification rates and gross NO_3_-N consumption rates in soils following imidacloprid applications after 90 days. Similarly, Simonin et al. [35] reported significant changes in extracellular enzyme activity and organic matter degradation without impacting plant or soil conditions in lab conditions. Findings from our study further support soil N concentrations were not significantly changed but did impact runoff concentrations.

### 3.4. Carbon Transport

DOC concentrations exported from each plot were normalized and modeled using a mixed ANOVA with a significance value of *p* < 0.05. Significant differences were not observed between treatments for DOC, which can be seen in Table 4. DOC load leaving the plots for all treatments and events was 19.87 ± 4.86 mg C/L by event, which was lower compared to a previous field plot study performed by Lentz and Westermann [31] that used a rototiller to disturb the soil, similar to this study. However, this was likely due to differences in soil amendments. The soil series used in this study was Maury Silty Loam with organic matter of 67.4 g kg^−1^, while Lentz and Westermann [31] used Portneuf silt loam soil with organic matter of 10 g kg^−1^ in addition to a manure amendment with 243 g kg^−1^ total C being added to all plots. However, significant differences in DOC concentrations exported by event were observed in this study (*p* < 0.0001) with events 11, 2, 1, 3, 5, 4, 9, and 6 having larger releases, with event 11 having the largest export (Supplemental Materials Figures S3 and S4). These observations were similar to a previous study by Royer et al. [36] who observed DOC concentrations increased following fertilizer applications including mineral fertilizer and liquid hog manure. Royer et al. [36] reported rototilling, residue incorporation, and rainfall intensity increased DOC concentrations in runoff. However, in our study we primarily observed implications of rototilling on DOC and nutrient release only during the first event following soil disruption. Nutrient and DOC release for all future events were significantly impacted by rainfall intensity (Figures S2 and S4).

### 3.5. Orthophosphate Transport

In contrast to C, the CuNI plots had significantly lower PO_4_-P concentrations released compared to other treatments (*p* < 0.0007) besides the C treatment, which was nearing significance (*p* = 0.0765). The Cu treatment was also close to significance compared to the C (*p* = 0.0626), but for higher releases of PO_4_-P. The average concentration of exported PO_4_-P was 1.37 ± 1.06 mg P/L higher in Cu, 1.32 ± 1.06 mg P/L higher in I, and 1.30 ± 1.07 mg P/L higher in NI, which were similar to reported values of Seo et al. [37]. Seo et al. [37] investigated fertilizer application methods, including localized compaction and doming, no-till broadcast, and no-till coulter injection, on sediment and nutrient losses through natural rainfall, similar to this study. Although this interaction with Cu and imidacloprid has not been investigated before, our study exhibits a significant impact to PO_4_-P runoff concentrations for these specific pesticide formulations for this soil series.

### 3.6. Physicochemical Properties of Runoff and Pumpkin Production

Runoff physicochemical properties were measured from collection pails following each rainfall event within 24 h. Significant differences between treatments were not observed for physicochemical properties, including water temperature, dissolved oxygen, specific conductivity, and water pH, but there was a significant difference between events for all properties (*p* < 0.0001). These observations were likely a combination of sampling time, rainfall volume and intensity, field cover conditions, and atmospheric conditions. Average water temperature of the runoff in the study was 23.9 ± 1.24 °C. Average specific conductivity of the runoff was 216.4 ± 395.1 µS/cm, while average pH of water was 6.74 ± 0.42 from the runoff of the plots. These observations were likely due to soil physicochemical properties resulting in slightly acidic level. Observations were similar to previous plot experiments investigating runoff quality impacts following fertilizer applications in the eastern United States [38,39]. Detailed observations from this study can be further reviewed in Supplementary Materials Tables S3 and S5. Further, it should be noted that significant differences in pumpkin production were not observed between any treatement in this study (Figure S5).

### 3.7. Copper (II) Hydroxide Transport

Copper (II) hydroxide was applied to plots following event 6, as directed by the pesticide label once foliage was established. Dissolved Cu was analyzed in runoff samples for events 3, 5, 6, 7, and 8. However, no significant differences in Cu concentration exported via runoff from each plot was observed following normalization and modeling using a mixed ANOVA with a significance value of *p* < 0.05. Visualization by treatments can be viewed in Supplementary Materials Figure S6 and Table S6. The average total Cu release across all events and treatments was 0.13 µg/m^2^, which was several magnitudes less than the minimum 0.26 mg/m^2^ seen in a previous study on copper (II) hydroxide by Rice et al. [40]. However, Rice et al. [40] applied commercially available conventional fungicide to crops with soil covers of vetch-mulch and polyethene while no soil cover was provided in this study. Further, fungicide was only applied once compared to three times in the Rice et al. [40] study. In addition, the field plots used in by Rice et al. [40] contained 1.3–1.6% organic carbon content in the soil and plots had an average slope of ~3–6%. In contrast, our study had soil organic matter ranging from 5.16 to 10.04% with slope gradients on average of 3%. The combination of these factors may have resulted in increased soil retention of copper when compared to Rice et al. [40].

Further, Rice et al. [40] observed volume of water in rainfall events had the greatest impact to copper load released from sites [40], which was corroborated Imfeld et al. [41]. However, Babcsányi et al. [42] using conventional Cu fungicide observed dissolved Cu concentrations ranging from 7.7 to 32.0 µg L^−1^, where in our study concentrations ranged from below detectable limits to 166 µg L^−1^ for the dissolved fraction. This could imply there is greater copper release because of nanopesticides; however, there was an estimated total recovered mass of 1.9 mg in this study from all plots, all 30 plots, where a mass of 0.624 g Cu was added from pesticide to a single plot meaning a total of 7.48 g were added to the plots. Further, Babcsányi et al. [42] using stable isotopes of varying Cu-based conventional fungicides and reported only 1% of applied Cu was exported through surface runoff and noted that most Cu was bound to clays and soil organic matter [42], which was similar to observations in our study.

**Figure 4:**
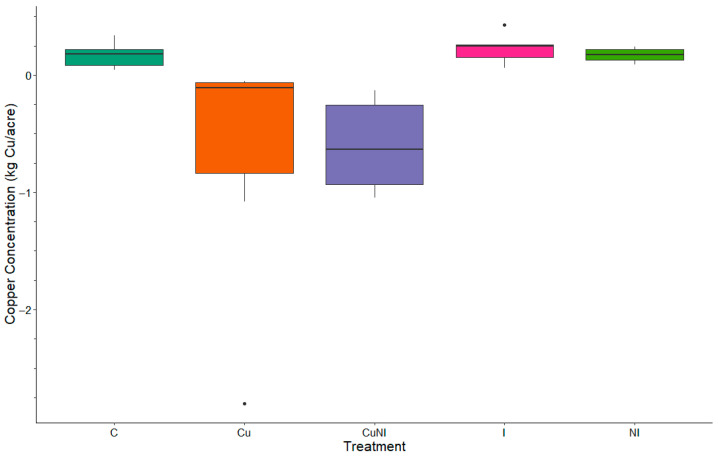
Box plot of total copper loss by treatment (mass[kg/acre], n = 5) between pre-applications at the beginning of the growing season and end of the growing season by treatment (n = 5).

The total Cu observed in soils in our study did have significant differences (*p* = 0.0068) between treatments, with plots receiving copper (II) hydroxide (Cu and CuNI) gaining 1.55 and 1.32 kg ha^−1^, respectively, retaining majority of the copper applied, in the top 20 cm of the soil. However, other plots lost total Cu in from the top 20 cm soil (Figure 4). These observations were similar to Wightwick et al. [43], where vineyards with high Cu pesticide applications were assessed and reported to have an increase in soil Cu concentrations compared to local areas. Soil Cu toxicity to soil invertebrates and crops has been observed to vary with soil properties [44]. For example, observed in Criel et al. [44] evaluated varying concentrations of soil Cu to identify toxicity levels for invertebrates in a variety of soils. Similarly, Zhang et al. [45] evaluated varied concentrations of soil Cu and cadmium (Cd) to identify toxicity levels for barley in a variety of soils. However, in Zhang et al. [45] did not approach the critical values for Cu toxicity, 8.27 to 50.13 mg kg^−1^, in soil following one year with concentrations averaging 2.03 mg kg^−1^ in applied plots. Although our observations of were well below chronic concentrations identified [44,45], future field studies should investigate environmental implications following prolonged application and exposure to these nanopesticides.

## 4. Conclusions

The fate, transport, and environmental impacts of copper (II) hydroxide, individually and combined with nano-imidacloprid were evaluated in this study. Nanoformulations of copper (II) hydroxide and its interactions with another nanopesticide, imidacloprid, were observed to lower N and P species export significantly from field plots. Specifically, significantly lower concentrations of NH_4_-N, PO_4_-P, and TN in the surface runoff for treatments of CuNI were observed. However, the application of copper (II) hydroxide was not observed to impact runoff concentration of Cu, but increased Cu in the soil, similar to previous observations of bulk Cu applications. These observations emphasize the importance of soil health management to avoid Cu buildup in agricultural soils. Further, based on observations from this study site and the use of these pesticide formulations, dual pesticide uses impacted N and P species in runoff compared to utilizing only one pesticide or the absence of pesticides. Future considerations to investigate the impacts on transport of these pesticides into groundwater and plant uptake along with long-term implications to soil health and plant uptake should be considered.

## Figures and Tables

**Table 1:** Mass of applied pesticides and nutrients in pesticide mixture per plot using manufacturer recommended application rates.

Treatment	Imidacloprid per Plot (g)	Cu per Plot (g)	N per Plot (g)
Cu	0	0.624	0
CuNI	0.632	0.624	0.172
I	2.67	0	0.732
NI	0.632	0	0.172
C	0	0	0

**Table 2:** Precipitation event date, intensity, and depth during 2022 field sampling.

Event	1	2	3	4	5	6	7	8	9	10	11
Date	July 9	July 18	July 26	July 28	July 31	August 1	August 5	August 6	August 8	August 11	August 30
Rainfall Depth (mm)	13 *	13.5 *	20.2	10.6	32.6	28.4	7.4	8.2	15	14.8	27.4
Rainfall Intensity (mm/hr)	-	-	28	57	42.4	52	28	6.5	42	36	57

* Values were collected from local NOAA station.

**Table 3:** Runoff Concentrations of NO_3_-N, NH_4_-N, TKN, and TN by Treatment in mg/L with grouping.

Treatment	NO_3_-N		NH_4_-N		TKN		TN	
	Mean (Std)	Range	Mean (Std)	Range	Mean (Std)	Range	Mean (Std)	Range
Cu	3.45 (5.96) ^a^	0.02–63.4	2.36 (4.65) ^ab^	0.02–26.9	4.67 (3.07) ^a^	0.64–54.8	12.60 (2.38) ^a^	1.42–80.1
CuNI	2.81 (4.69) ^a^	0.04–72.2	2.20 (6.71) ^b^	0.02–39.1	3.86 (3.09) ^a^	0.54–48.5	9.86 (2.42) ^b^	1.13–77.6
I	2.77 (5.20) ^a^	0.04–103.0	2.07 (7.39) ^ab^	0.02–51.0	4.09 (3.23) ^a^	0.09–33.75	10.56 (2.19) ^ab^	1.98–111.8
NI	2.29 (5.71) ^a^	0.03–88.7	3.18 (5.92) ^ab^	0.02–48.2	4.07 (2.81) ^a^	0.70–48.3	9.34 (2.53) ^ab^	1.78–95.6
C	3.49 (5.59) ^a^	0.04–43.6	3.65 (6.10) ^a^	0.02–83.9	4.20 (3.89) ^a^	0.04–55.3	11.14 (2.83) ^ab^	0.50–67.0
Overall	2.94 (5.39)	0.02–103.0	2.62 (6.12)	0.02–83.9	4.17 (3.20)	0.04–55.3	10.69 (2.47)	0.50–111.8

(^a,b^) Tukey honest significance difference groups determined at *p* < 0.05 level.

**Table 4:** Runoff concentrations of PO_4_-P and DOC by treatment.

Treatment	PO_4_-P (mg/L)		DOC (mg/L)	
	Mean (Std)	Range	Mean (Std)	Range
Cu	2.18 (2.36) ^a^	0.32–14.2	23.89 (4.20) ^a^	0.03–531.6
CuNI	1.58 (2.74) ^b^	0.13–11.6	18.69 (4.63) ^a^	0.03–351.4
I	1.98 (2.34) ^a^	0.53–15.6	14.96 (6.63) ^a^	0.03–299.7
NI	1.92 (2.29) ^a^	0.63–16.9	22.28 (3.08) ^a^	0.03–781.3
C	1.89 (2.58) ^ab^	0.31–20.8	21.44 (5.76) ^a^	0.03–547.7
Overall	1.90 (2.47)	0.13–20.8	19.88 (4.86)	0.03–781.3

(^a,b^) Tukey honest significance difference groups determined at *p* < 0.05 level.

## Data Availability

The original contributions presented in this study are included in the article/supplementary material. Further inquiries can be directed to the corresponding author.

## Supplementary Materials

The following supporting information can be downloaded at: https://www.mdpi.com/article/10.3390/toxics13090758/s1, Figure S1: a. Nitrate-Nitrate, b. Ammonium, c. TKN, and d. TN box and whisker plots by treatment (ln(concentration[mg/L])); Figure S2: a. NO_3_-N, b. NH_4_-N, c. TKN-N, and d. TN overall average by event and treatment (ln(concentration[mg/L])); Figure S3: a. Phosphate and b. Dissolved organic carbon overall average by treatment (ln(concentration[mg/L])); Figure S4: a. Phosphate and b. Dissolved organic carbon overall average by event treatment (ln(concentration[mg/L])); Figure S5: Box plot of pumpkin weight by treatment (kg); Figure S6: Box plot of runoff copper loading (a.) by treatment and (b.) by treatment and event (ln(concentration[µg/L])); Table S1: Initial soil conditions by each plot; Table S2: Instrument Conditions of ICP-MS; Table S3: Average temperature (°C) and standard deviation (SD) for each treatment by event. Temperature data was collected in the pails by YSI EXO multiparameter sonde; Table S4: Average specific conductivity (µS/cm) and standard deviation (SD) for each treatment by event. Specific conductivity data was collected in the pails by YSI EXO multiparameter; Table S5: Average pH and standard deviation (SD) for each treatment by event. The pH data was collected in the pails by YSI EXO multiparameter sonde; Table S6: Average total copper mass (µg) and standard deviation (SD) for each treatment by event.
