# Supplementary material for: Implications of Nutrient Fate and Transport Following Nanopesticide Applications in Agricultural Field Plots in Central Kentucky
Source: Toxics. 2025 Sep 6;13(9):758. doi: 10.3390/toxics13090758 (PMC12474015; doi:10.3390/toxics13090758)
Supplement: Supplementary file 1 [file toxics-13-00758-s001.zip › toxics-3733430-supplementary.pdf]

Supplementary Materials

Implications of Nutrient Fate and Transport Following Nanopesticide Applications in Agricultural Field Plots in Central Kentucky

William Rud [1,*], Manuel D. Montaño [2], Daniel N. Miller [3], Wayne Sanderson [1], Carmen Agouridis [1], Brianna F. Benner [2] and Tiffany L. Messer [1]

[1] Biosystems and Agricultural Engineering Department, University of Kentucky, 128 Barnhart, Lexington, KY 40506, USA
[2] Department of Environmental Sciences, Western Washington University, Bellingham, WA 98225, USA
[3] United States Department of Agriculture, Agricultural Research Service, Lincoln, NE 68583, USA
* Correspondence: william.rud@uky.edu

Supplemental Material

Figure S1 – Figure S6
Table S1 – Table S6

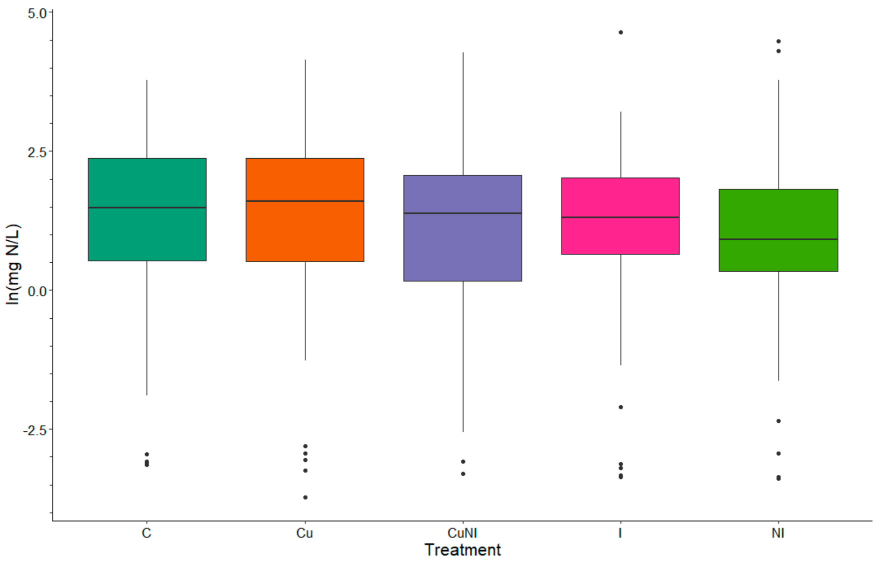
5.0
2.5
0.0
-2.5
ln(mg N/L)
C
Cu
CuNI
I
NI
Treatment

a

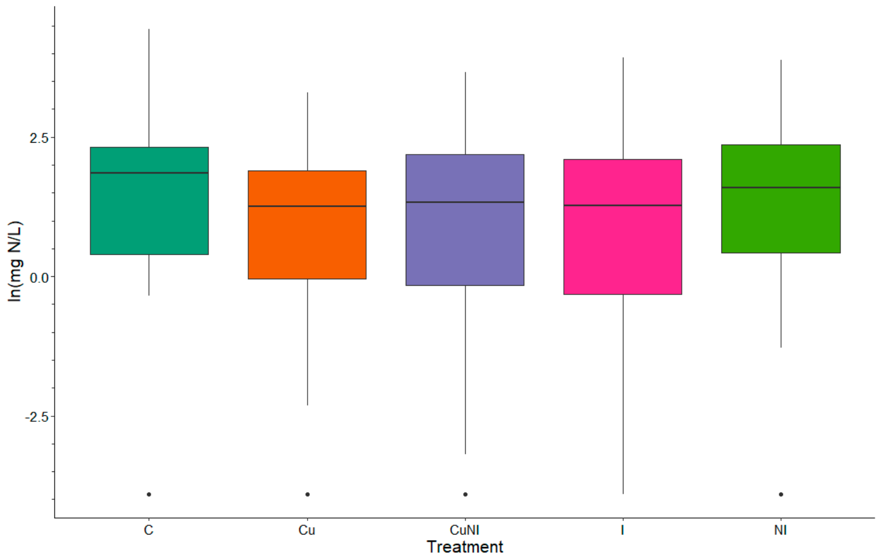
2.5
0.0
-2.5
ln(mg N/L)
C
Cu
CuNI
I
NI
Treatment

b.

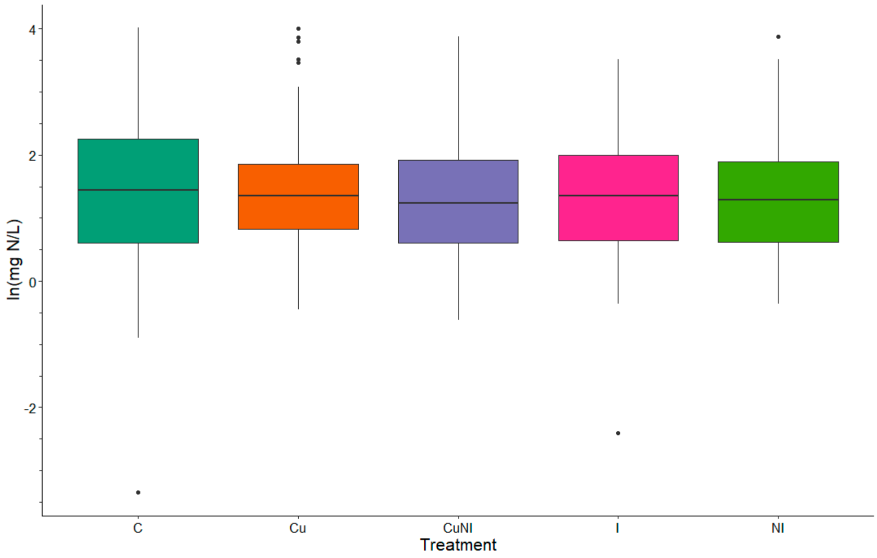

c

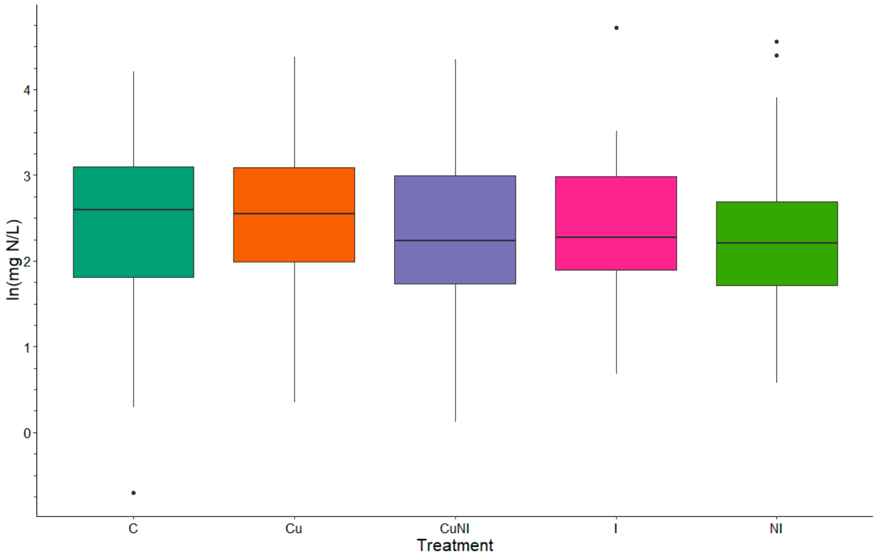

d.

Figure S1: a. NO_3-N, b. NH_4-N, c. TKN-N, and d. TN box and whisker plots by treatment (ln(concentration[mg/L])).

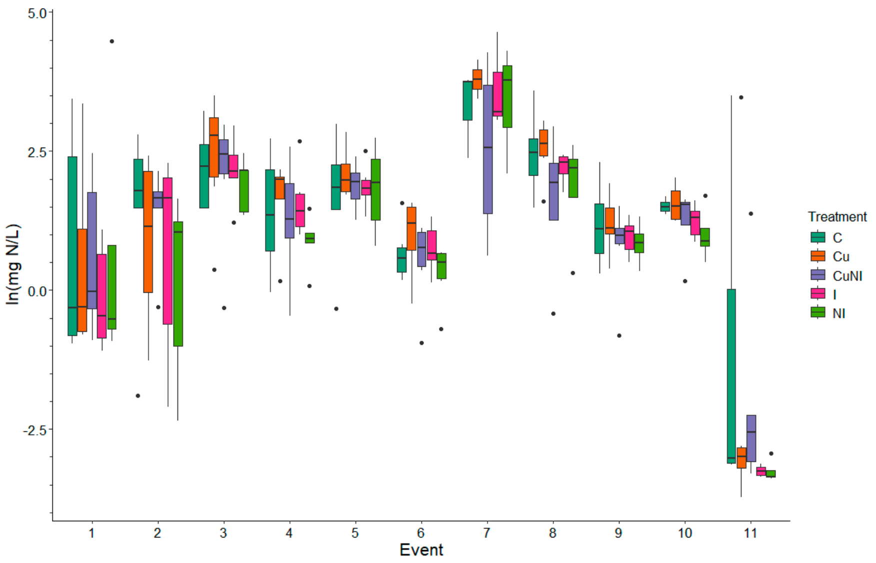
5.0
2.5
0.0
-2.5
ln(mg N/L)
1
2
3
4
5
6
7
8
9
10
11
Event
Treatment
C
Cu
CuNI
I
NI

a

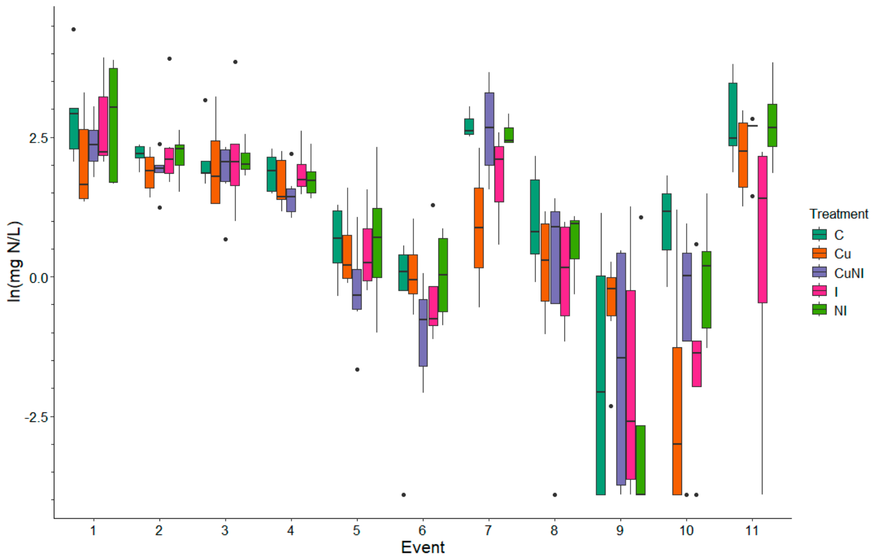
2.5
0.0
-2.5
ln(mg N/L)
1
2
3
4
5
6
7
8
9
10
11
Event
Treatment
C
Cu
CuNI
I
NI

b

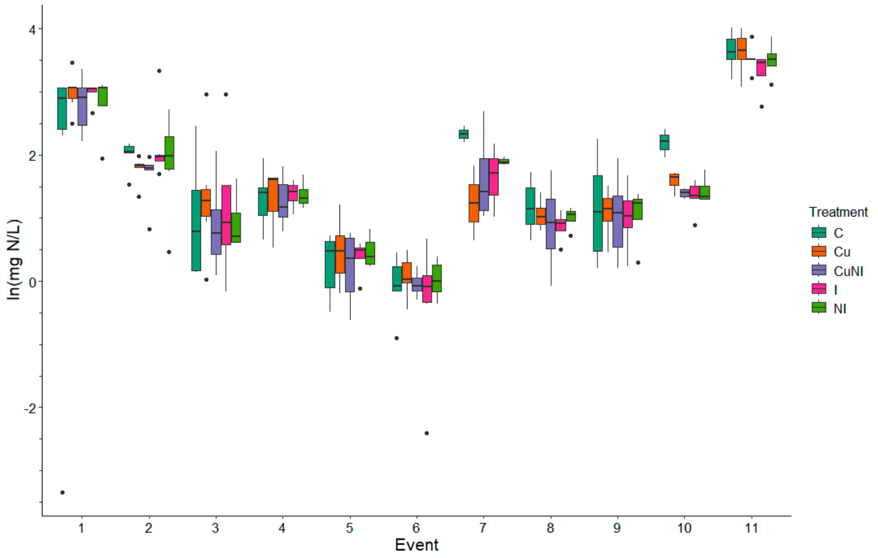

c

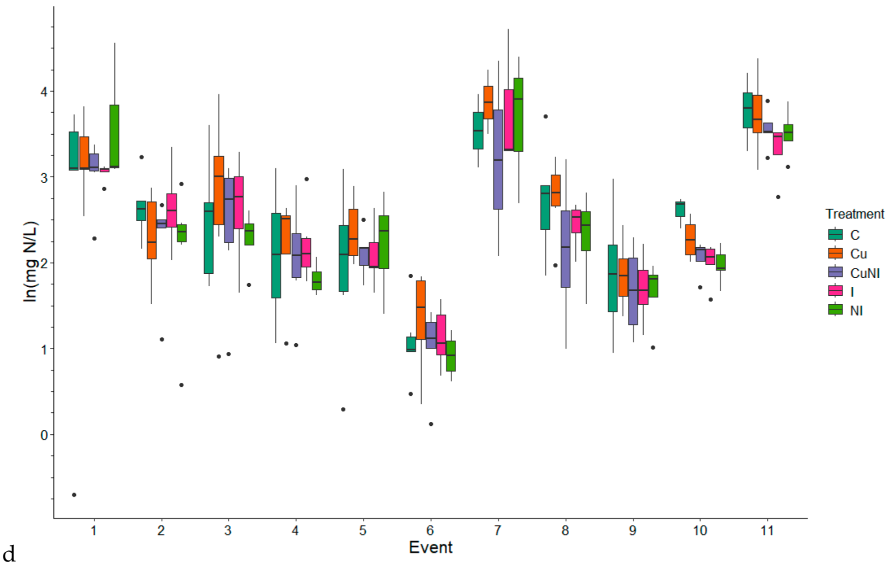

d

Figure S2: a. NO_3-N, b. NH_4-N, c. TKN-N, and d. TN overall average by event and treatment (ln(concentration[mg/L])).

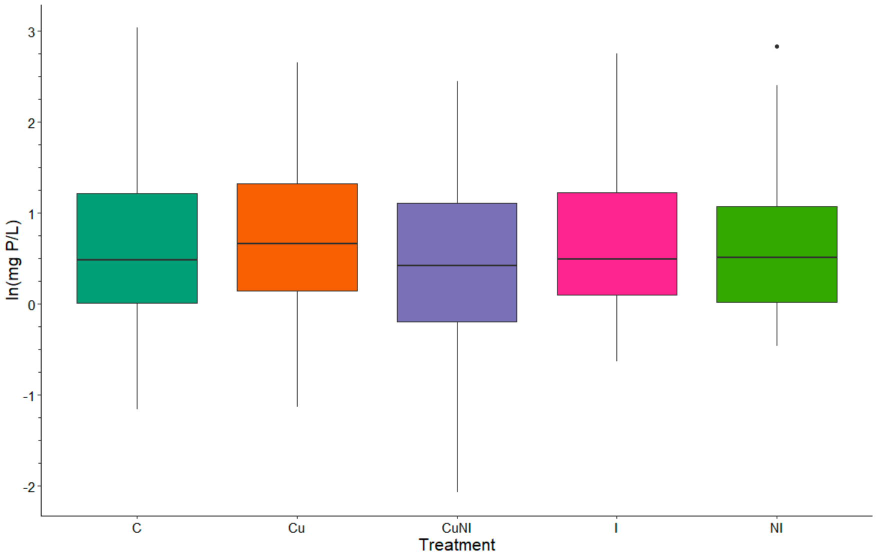

a

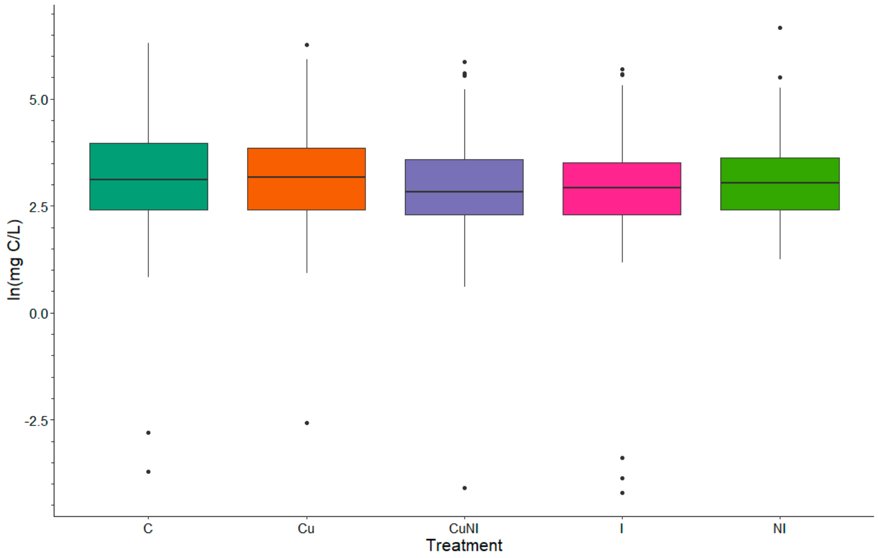

b

Figure S3: a. Phosphate-P and b. Dissolved organic carbon overall average by treatment (ln(concentration[mg/L])).

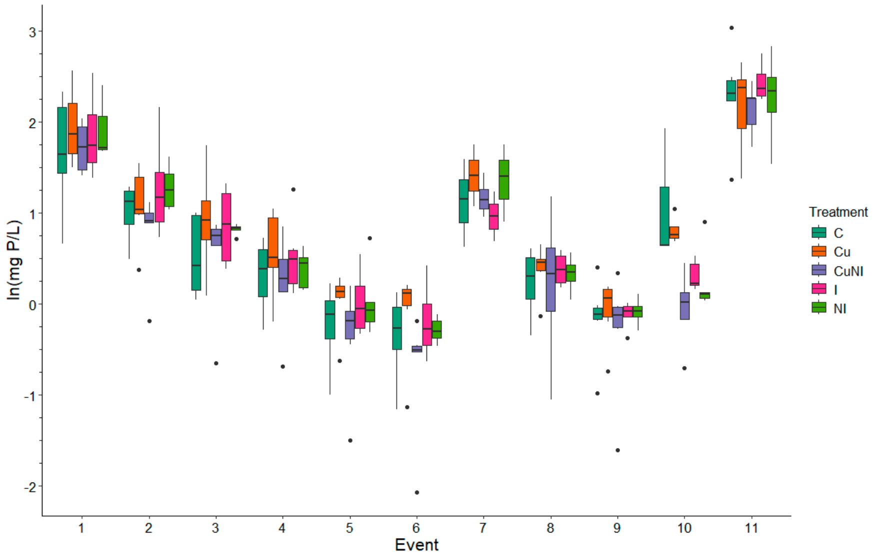

a

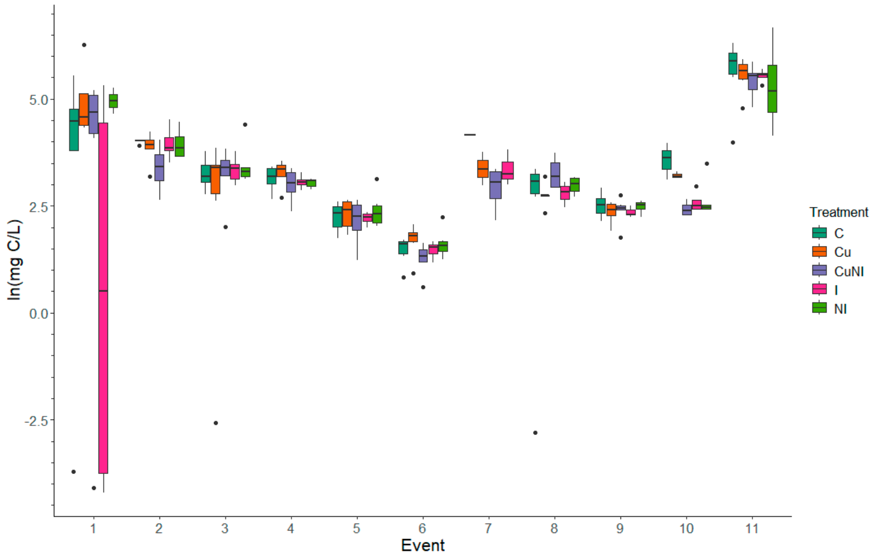

b

Figure S4: a. Phosphate-P and b. Dissolved organic carbon overall average by event treatment (ln(concentration[mg/L])).

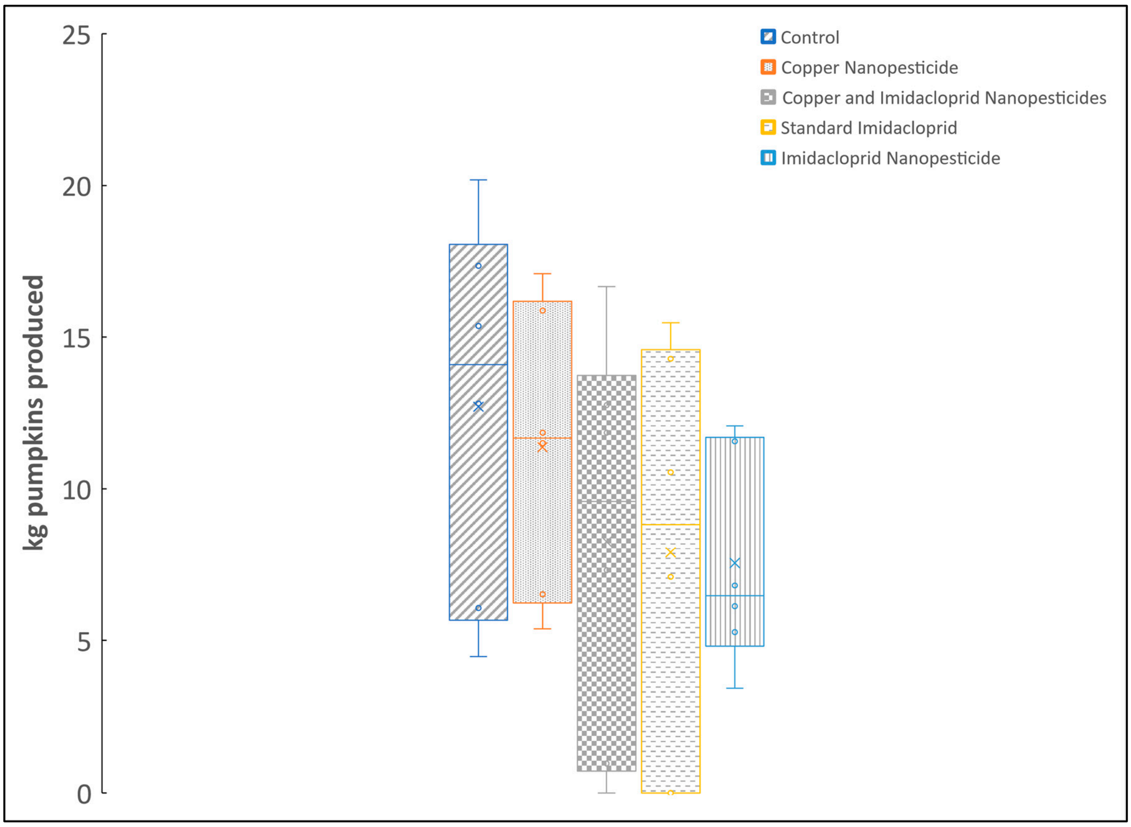

Figure S5: Box plot of pumpkin weight by treatment (kg).

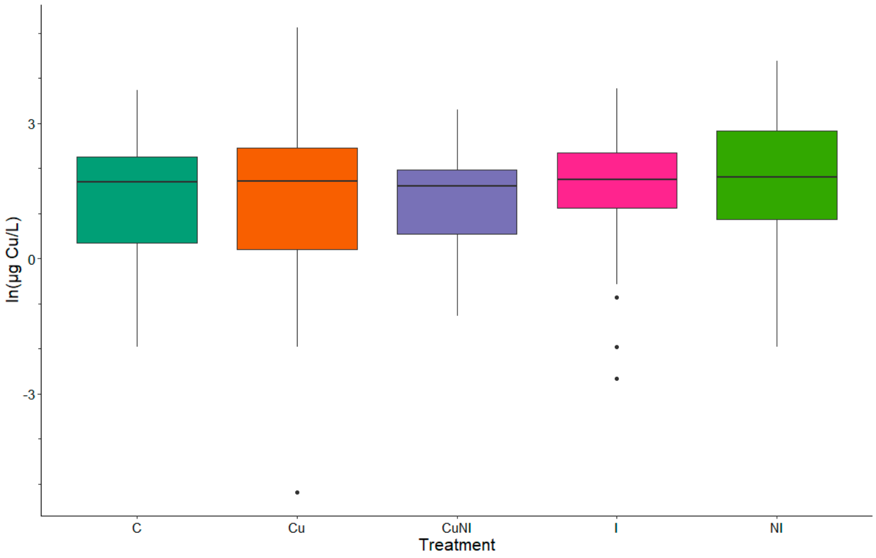

a

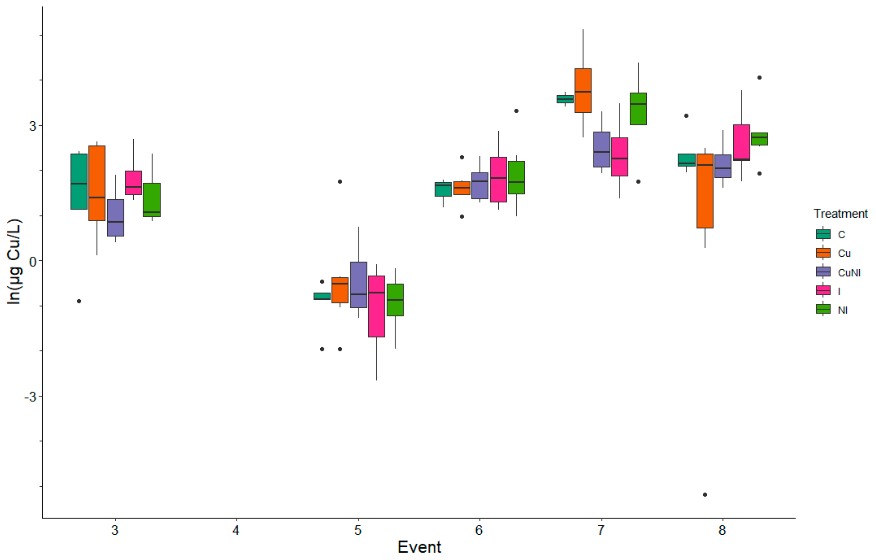

b

Figure S6: Box plot of runoff copper loading (a.) by treatment and (b.) by treatment and event (ln(concentration[µg/L])).

Table S1: Initial soil conditions by each plot

Plot	Treatment	Date	1 M KCL soil pH	Sikora II Buffer pH	P (lbs/a)	K(lbs/a)	Ca (lbs/a)	Mg (lbs/a)	Zn (lbs/a)	Plant Available Water	Field Capacity Water	Wilting Point Water
B1	CuNI	Monday, March 14, 2022	5.49	6.71	261	992	3615	631	31.2	38.32%	59.71%	21.38%
B2	Cu	Monday, March 14, 2022	6.01	7.01	218	935	4564	523	19.4	33.15%	52.77%	19.62%
B3	NI	Monday, March 14, 2022	5.49	6.75	259	1097	4037	715	20.4	35.22%	56.54%	21.32%
B4	C	Monday, March 14, 2022	5.13	6.57	257	941	3497	672	92.4	36.94%	61.43%	24.49%
B5	I	Monday, March 14, 2022	5.7	6.9	279	1293	3555	615	22	34.44%	57.93%	23.48%
B6	NI	Monday, March 14, 2022	5.2	6.61	238	1091	3355	472	12.1	35.90%	55.30%	19.40%
B7	CuNI	Monday, March 14, 2022	5.35	6.7	266	914	4349	670	30.8	39.02%	62.89%	23.87%
B8	C	Monday, March 14, 2022	5.26	6.62	184	889	3560	800	18.7	33.84%	54.79%	20.95%
B9	I	Monday, March 14, 2022	5.11	6.69	201	1105	3131	657	66.8	34.38%	54.95%	20.57%
B10	Cu	Monday, March 14, 2022	5.37	6.67	210	1194	3973	713	25.6	30.62%	52.23%	21.62%
C1	NI	Monday, March 14, 2022	5.66	6.91	227	1079	3895	482	45.6	31.27%	49.44%	18.17%
C2	C	Monday, March 14, 2022	5.31	6.73	223	1021	3485	504	10.8	24.56%	43.29%	18.47%
C3	I	Monday, March 14, 2022	5.32	6.75	346	867	4184	633	27.6	25.58%	46.90%	21.32%
C4	Cu	Monday, March 14, 2022	5.23	6.69	282	922	3915	578	24.5	24.21%	44.83%	20.63%
C5	CuNI	Monday, March 14, 2022	5.39	6.82	209	1036	3432	419	11.1	22.84%	41.70%	18.87%
C6	C	Monday, March 14, 2022	5.42	6.7	258	1414	3852	581	32.9	25.72%	46.58%	20.86%
C7	I	Monday, March 14, 2022	5.39	6.82	294	1206	4025	611	27.6	27.57%	50.03%	22.46%
C8	Cu	Monday, March 14, 2022	5.28	6.6	302	872	4017	782	28.2	28.26%	52.04%	23.78%
C9	NI	Monday, March 14, 2022	5.25	6.75	280	1176	3596	666	24.1	27.16%	48.57%	21.41%
C10	CuNI	Monday, March 14, 2022	5.24	6.6	233	981	3787	631	101	27.92%	49.14%	21.22%
D1	I	Monday, March 14, 2022	5.64	6.84	226	875	3545	449	31.9	27.27%	47.40%	20.13%
D2	CuNI	Monday, March 14, 2022	5.48	6.74	322	959	3784	752	43.3	29.05%	49.61%	20.56%
D3	Cu	Monday, March 14, 2022	5.19	6.55	291	956	3478	687	31.1	31.92%	53.90%	21.98%
D4	C	Monday, March 14, 2022	5.42	6.7	282	1039	3727	634	94	26.84%	49.07%	22.23%
D5	NI	Monday, March 14, 2022	5.13	6.63	231	956	3290	505	14.5	25.99%	43.53%	17.54%
D6	Cu	Monday, March 14, 2022	5.38	6.68	269	979	3700	627	89.4	27.17%	46.62%	19.46%
D7	NI	Monday, March 14, 2022	5.04	6.5	301	938	3069	707	61.7	29.77%	49.82%	20.04%
D8	CuNI	Monday, March 14, 2022	4.96	6.45	227	850	3088	654	49.3	30.55%	50.47%	19.92%
D9	I	Monday, March 14, 2022	4.82	6.41	210	806	2743	555	44.3	28.21%	46.85%	18.64%
D10	C	Monday, March 14, 2022	4.73	6.41	206	645	2807	492	42.9	26.74%	44.80%	18.06%

Table S1: (continued)

Boron (lbs/a)	Soil Organic Matter	Total N	Meh3_Cu (lbs/a)	Meh3_Mn (lbs/a)	Soil Texture	Sand	Silt	Clay	Meh3_Fe (lbs/a)	Cation Exchange Capacity (meq/100g)	Base Saturation	Exchange K (meq/100g)	Exchange Ca (meq/100g)
1	8.34%	0.467%	3.16	408	silt loam	15.99%	71.30%	12.72%	372	20.64	89.21%	1.56	13.4
0.52	6.26%	0.338%	2.92	466	silt loam	15.36%	69.13%	15.52%	368	20.08	98.74%	1.4	15.5
0.96	8.70%	0.482%	2.46	300	silt loam	16.83%	68.27%	14.90%	334	21.05	111.68%	1.89	17.6
0.88	10.04%	0.553%	1.98	230	silt loam	18.35%	65.97%	15.68%	302	20.8	81.01%	1.65	11.3
0.68	7.19%	0.421%	2.24	276	silt loam	16.56%	69.31%	14.14%	318	20.08	86.43%	2.07	11.8
0.84	8.20%	0.445%	2.3	306	silt loam	17.69%	69.30%	13.01%	340	20.01	77.38%	1.66	11.3
0.84	8.17%	0.432%	2.82	500	silt loam	15.92%	69.63%	14.46%	420	22.32	83.06%	1.48	13.6
0.56	5.81%	0.334%	2.28	352	silt loam	13.96%	69.45%	16.59%	356	20.64	79.49%	1.14	11.3
0.76	6.30%	0.359%	2.36	398	silt loam	14.16%	70.48%	15.36%	372	19.39	80.33%	1.66	10.3
0.68	5.62%	0.332%	2.7	360	silt loam	13.46%	66.62%	19.92%	364	20.4	85.89%	1.87	12.1
0.52	5.54%	0.320%	2.24	266	silt loam	13.20%	71.68%	15.12%	292	17.24	87.36%	1.55	11.3
1.52	5.18%	0.297%	2.54	300	silt loam	13.04%	72.07%	14.89%	322	16.52	78.26%	1.48	9.2
1.92	6.57%	0.396%	3.88	254	silt loam	13.43%	72.37%	14.20%	366	20.32	87.21%	1.3	13.2
2.28	9.13%	0.538%	2.7	256	silt loam	16.86%	69.32%	13.82%	310	20.01	81.59%	1.29	12.1
1.72	6.57%	0.392%	2.44	272	silt loam	14.10%	71.84%	14.06%	272	17.72	97.98%	1.62	13.6
1.36	5.26%	0.299%	2.42	210	silt loam	13.47%	71.41%	15.12%	324	19.01	82.44%	1.93	11
1.52	6.55%	0.390%	2.64	264	silt loam	14.06%	71.76%	14.18%	318	21.63	88.91%	1.93	13.8
1.2	6.59%	0.373%	3.04	328	silt loam	14.13%	69.13%	16.74%	406	21.22	83.16%	1.38	12.3
1.52	6.81%	0.385%	2.58	334	silt loam	14.58%	70.80%	14.62%	372	20.01	85.56%	1.86	11.6
1.6	7.21%	0.415%	2.14	268	silt loam	15.12%	70.06%	14.82%	338	20.08	82.04%	1.56	11.7
1.2	6.28%	0.377%	2.7	282	silt loam	15.27%	72.35%	12.38%	304	19.25	97.48%	1.65	14.3
0.84	5.16%	0.323%	3.5	274	silt loam	12.10%	73.52%	14.38%	360	20.85	87.29%	1.54	13
1.12	6.74%	0.414%	2.6	228	silt loam	14.70%	75.01%	10.29%	350	22.14	85.67%	1.74	13.4
1.12	6.62%	0.400%	3	246	silt loam	15.09%	72.49%	12.42%	324	21.53	86.83%	1.99	13.4
0.92	4.85%	0.288%	2.42	298	silt loam	13.30%	72.44%	14.26%	322	16.54	78.74%	1.48	9.44
0.84	6.88%	0.406%	2.88	294	silt loam	14.93%	73.34%	11.73%	338	19.48	85.95%	1.62	11.8
0.88	6.78%	0.395%	3.62	280	silt loam	16.55%	72.96%	10.49%	340	19.8	81.96%	1.46	11.1
0.92	6.21%	0.375%	2.46	266	silt loam	15.53%	74.09%	10.37%	354	19.02	80.62%	1.35	10.7
0.96	6.52%	0.392%	2.46	254	silt loam	15.45%	72.88%	11.66%	332	19.25	76.54%	1.64	10.1
1.12	6.12%	0.367%	2.44	248	silt loam	13.34%	74.78%	11.89%	346	17.7	70.85%	1.08	9.21

Table S1: (continued)

Exchange Mg (meq/100g)	Exchange Na (meq/100g)	Calculated soil-water pH
3.44	0.01	6.34
2.91	0.02	6.81
4	0.01	6.34
3.88	0.02	6.01
3.47	0.01	6.53
2.5	0.01	6.07
3.44	0.02	6.21
3.95	0.02	6.13
3.6	0.01	5.99
3.54	0.02	6.23
2.21	0.01	6.49
2.24	0.01	6.17
3.21	0.01	6.18
2.93	0.01	6.1
2.14	0.01	6.24
2.73	0.01	6.27
3.49	0.02	6.24
3.95	0.02	6.14
3.64	0.02	6.12
3.21	0.01	6.11
2.8	0.01	6.47
3.65	0.01	6.33
3.82	0.01	6.06
3.29	0.02	6.27
2.09	0.01	6.01
3.31	0.02	6.24
3.65	0.01	5.93
3.28	0.01	5.85
2.98	0.02	5.73
2.24	0.01	5.64

Table S2: *Instrument Conditions of ICP-MS*

ICP-MS (Agilent 7500ce)	
Instrument Parameter	Value
Nebulizer Gas Flow	0.81 ml/min
Sample Flow Rate	0.34 ml/min
Spray Chamber	Scott Double Pass
ICP RF Power	1500 W
Dwell time	100 ms
Analyte Elements	^{9}Be, ^{27}Al, ^{51}V, ^{52}Cr, ^{55}Mn, ^{59}Co, ^{60}Ni, ^{63}Cu, ^{66}Zn, ^{75}As, ^{78}Se, ^{95}Mo, ^{111}Cd, ^{123}Sb, ^{205}Tl, ^{208}Pb, ^{232}Th, ^{238}U
Internal Standard Elements	^{45}Sc, ^{115}In, ^{159}Tb, ^{209}Bi

Table S3: Average temperature (°C) and standard deviation (SD) for each treatment by event. Temperature data was collected in the pails by YSI EXO multiparameter sonde.

Event	Control		Copper Nanopesticide		Nano-imidacloprid		Copper Nanopesticide Nanoimidacloprid		Conventional Imidacloprid	
	Mean	SD	Mean	SD	Mean	SD	Mean	SD	Mean	SD
1	-	-	-	-	-	-	-	-	-	-
2	22.74	0.23	23.00	0.25	23.13	0.16	23.12	0.24	23.43	0.58
3	24.30	0.39	24.18	0.29	24.54	0.62	24.5	0.60	24.35	0.25
4	25.15	0.51	25.38	0.59	25.94	1.18	25.30	1.02	25.93	0.69
5	22.25	0.21	22.32	0.23	22.88	0.72	22.42	0.51	22.90	0.61
6	24.05	0.46	24.37	0.38	24.22	0.77	23.93	0.18	23.98	0.49
7	-	-	-	-	-	-	-	-	-	-
8	23.60	23.88	24.87	0.22	21.17	0.12	20.9	0.18	21.18	0.19
9	23.88	0.34	23.98	0.29	24.50	0.54	24.63	0.86	24.73	0.68
10	-	-	-	-	24.48	0.30	24.33	0.31	25.18	0.68
11	24.43	0.41	24.15	0.34	24.47	0.57	24.23	0.45	24.23	0.47

Table S4: Average specific conductivity (µS/cm) and standard deviation (SD) for each treatment by event. Specific conductivity data was collected in the pails by YSI EXO multiparameter sonde.

Event	Control		Copper Nanopesticide		Nano-imidacloprid		Copper Nanopesticide Nanoimidacloprid		Conventional Imidacloprid	
	Mean	SD	Mean	SD	Mean	SD	Mean	SD	Mean	SD
1	-	-	-	-	-	-	-	-	-	-
2	187.56	55.9	197.52	75.68	183.87	59.11	164.52	57.95	229.08	111.44
3	132.26	71.23	158.86	61.20	117.0	39.13	138.58	61.34	138.35	44.03
4	104.80	26.6	131.84	53.98	81.03	11.89	76.92	27.26	91.72	14.27
5	73.48	24.57	80.77	28.93	56.28	12.66	58.10	21.82	63.75	8.16
6	28.93	13.68	44.73	14.82	32.66	5.11	26.98	8.43	33.18	5.88
7	-	-	-	-	-	-	-	-	-	-
8	136.40	55.58	130.92	47.84	116.5	25.14	99.70	38.52	108.65	12.91
9	78.38	23.96	80.47	26.78	74.5	13.00	63.48	27.17	68.57	23.22
10	-	-	-	-	101.0	24.37	89.60	24.85	86.36	27.0
11	1546	977	1014	326	1336	5583	1184	248	1112	223.83

Table S5: Average pH and standard deviation (SD) for each treatment by event. The pH data was collected in the pails by YSI EXO multiparameter sonde.

Event	Control		Copper Nanopesticide		Nano-imidacloprid		Copper Nanopesticide Nanoimidacloprid		Conventional Imidacloprid	
	Mean	SD	Mean	SD	Mean	SD	Mean	SD	Mean	SD
1	-	-	-	-	-	-	-	-	-	-
2	6.63	0.17	6.69	0.16	6.71	0.06	6.66	0.11	6.71	0.13
3	6.64	0.08	6.64	0.15	6.61	0.06	6.60	0.12	6.58	0.19
4	6.70	0.10	6.87	0.19	6.78	0.17	6.73	0.24	6.73	0.09
5	6.48	0.11	6.59	0.10	6.58	0.17	6.49	0.10	6.54	0.14
6	6.31	0.14	6.54	0.18	6.40	0.12	6.30	0.17	6.29	0.14
7	-	-	-	-	-	-	-	-	-	-
8	7.14	0.20	7.04	0.20	6.95	0.46	7.35	0.81	6.85	0.11
9	6.61	0.25	6.42	0.14	6.43	0.06	6.51	0.11	6.41	0.10
10	7.04	0.32	7.09	0.26	6.65	0.13	6.63	0.10	6.49	0.20
11	7.77	0.34	7.67	0.26	7.50	0.54	7.89	0.14	7.86	0.14

Table S6: Average total copper mass (μg) and standard deviation (SD) for each treatment by event.

Event	Control		Copper Nanopesticide		Nano-imidacloprid		Copper Nanopesticide Nanoimidacloprid		Conventional Imidacloprid	
	Mean	SD	Mean	SD	Mean	SD	Mean	SD	Mean	SD
1	-	-	-	-	-	-	-	-	-	-
2	-	-	-	-	-	-	-	-	-	-
3	5.65	7.47	35.37	33.26	5.34	9.88	4.26	11.20	10.30	13.35
4	-	-	-	-	-	-	-	-	-	-
5	2.62	2.29	10.15	14.87	1.67	1.29	6.77	7.09	3.63	3.07
6	28.75	16.06	39.57	16.59	26.14	18.97	37.65	16.06	41.45	43.85
7	1.21	1.91	3.61	3.55	2.91	3.99	2.02	2.36	1.14	1.28
8	12.07	9.43	10.00	8.37	14.89	16.45	6.95	8.55	13.36	15.19
9	-	-	-	-	-	-	-	-	-	-
10	-	-	-	-	-	-	-	-	-	-
11	-	-	-	-	-	-	-	-	-	-
